# Premature Mortality Due to Malignancies of the Kidney and Bladder in Japan, 1980–2010

**DOI:** 10.2188/jea.JE20180140

**Published:** 2019-12-05

**Authors:** Truong-Minh Pham, Tatsuhiko Kubo, Yoshihisa Fujino, Naohiro Fujimoto, Ikko Tomisaki, Akinori Minato, Shinya Matsuda

**Affiliations:** 1 Surveillance and Reporting, Cancer Control Alberta, Alberta Health Services, Edmonton, Alberta, Canada; 2 Department of Preventive Medicine and Community Health, School of Medicine, University of Occupational and Environmental Health, Kitakyushu, Japan; 3 Department of Environmental Epidemiology, Institute of Industrial Ecological Sciences, University of Occupational and Environmental Health, Kitakyushu, Japan; 4 Department of Urology, School of Medicine, University of Occupational and Environmental Health, Kitakyushu, Japan

**Keywords:** kidney cancer, bladder cancer, years of life lost, average years of life lost, average lifespan shortened

## Abstract

**Background:**

In the present study, we examined the trends of premature mortality due to kidney and bladder cancers among the Japanese population from 1980 through 2010.

**Methods:**

Mortality data were obtained from the World Health Organization mortality database. Years of life lost (YLL) was estimated using Japanese life tables. Average lifespan shortened (ALSS) was calculated and defined as the ratio of years of life lost relative to the expected lifespan.

**Results:**

Over the study period, the age-standardized rates to the World Standard Population for deaths from kidney and bladder cancers were stable. The average years of life lost (AYLL) measure shows decreases of about 4 and 6 years of life for kidney cancer in men and women, respectively, and decreases of about 2 years of life for bladder cancer in both sexes. The ALSS shows that patients with kidney cancer lost 21.0% and 24.7% of their lifespan among men and women in 1980, whereas respective losses were 15.3% and 15.8% in 2010. Also, patients with bladder cancer on average lost 13.5% in men and 14.2% in women in 1980 and 10.8% in men and 11.1% in women in 2010.

**Conclusions:**

Our study shows favorable trends in premature mortality for kidney and bladder cancers in Japan over a 30-year period; however, patients with bladder cancer on average lost a smaller proportion of their lifespan compared to those with kidney cancer. The development of a novel ALSS measure is convenient in examination of the burden of premature mortality over time.

## INTRODUCTION

Malignancies of the kidney and bladder account for the majority of cancers of the urinary system, which consists of the kidneys, bladder, upper urinary tract (ureter and renal pelvis), and urethra. Japan generally has lower incidence rates of kidney and bladder cancers than western countries.^1^ A recent estimate, based on data from 11 population-based cancer registries in Japan for the year 2000, reported that kidney cancer accounted for about 2% of new cancers in both sexes, while bladder cancer accounted for 3% of new cancers in men and less than 2% in women.^2^

Although introduced before the 1990s, premature mortality in terms of years of life lost (YLL) has been under-utilized in the evaluation of the burden of chronic conditions. The YLL metric was initially based on subtraction of age at death from a fixed given age.^3^ The concept was thoroughly revised within the Global Burden of Disease (GBD) project, and the normative life expectancy^4^^–^^8^ was proposed to calculate YLL, rather than an established fixed age. On top of the discrepancy due to inconsistency between the aforementioned methods in YLL calculation, researchers have faced incomparability of “life lost” measures over time or across populations because the lifespan of the general population tends to get longer over time or life table structures are not similar between countries. Specifically, results from premature mortality of a particular health condition in the 2000s could not be compared to those in the 1980s because life expectancy improved with time. In order to improve comparability we proposed a new measure, the average lifespan shortened (ALSS), expressed as the ratio of years of life lost relative to expected lifespan according to national life tables. We have already demonstrated the utility of this novel measure in evaluating premature mortality from breast and the central nervous system cancers among the Canadian population.^9^^,^^10^

Given that little is known about premature mortality due to malignancies of the urinary tract in Japan, we conducted this study to examine the long-term trends of premature mortality due to kidney and bladder cancers among Japanese population over a long-term period from 1980 through 2010.

## METHODS

### Study population

In this study, we included malignancies of the kidney and bladder, which accounted for the majority of cancers of the urinary system. The mortality data for the Japanese population were obtained from the World Health Organization (WHO) mortality database^11^ for the period of 1980 through 2010. During this period, causes of death for Japanese data were coded according to the International Classification of Diseases and Injuries (ICD) 9^th^ Revision (ICD-9) from 1980 to 1994, and 10^th^ Revision (ICD-10) from 1995 onwards. Malignancies for kidney cancer were coded as 189.0 in the ICD-9 Revision and C64 in the ICD-10 Revision, and those for bladder cancer were coded as 188 in the ICD-9 Revision and C67 in the ICD-10 Revision.

### Statistical analyses

We calculated the age-standardized rates (ASRs) per 100,000 people for deaths due to kidney and bladder cancers according to the World Standard Population. We applied YLL computation according to the GBD Project.^4^^,^^5^ Briefly, YLL is calculated by multiplying, for each 5-year age group, the number of deaths with the age-specific life expectancy at the midpoint of the respective 5-year age group, as derived from life tables provided by the Ministry of Health, Labour and Welfare, Japan.^12^ Since there were several versions of life tables available over the study period, the following lifetables were used to calculate the YLL measure: the 1980 life tables for 1980–1984; the 1985 life tables for 1985–1989; the 1990 life tables for 1990–1994; the 1995 life tables for 1995–1999; the 2000 life tables for 2000–2004; and the 2005 life tables for 2005 and onward. Over the 30 year period, life expectancy at birth increased from 73 to 79 years in men and from 78 to 86 years in women.^12^ We then calculated the rate of YLL per 1,000 people adjusted to the World Standard Population to assess the burden of life loss relative to the entire population. The average years of life lost (AYLL) was estimated by dividing the total YLL by the total number of deaths in each calendar year. We calculated the ALSS, which was a ratio of years of life lost among people who died from kidney or bladder cancers compared to the expected lifespan based on the respective life tables. The below hypothetical examples demonstrate how the ALSS was calculated. A given man who died from bladder cancer in the year 1990 at his 60 years of age would have lost 20.0 years of life as indicates the 1990 Japanese male life table. So his lifespan was shortened by 25% resulting from his 20.0 years of life lost over the expected lifespan of 80.0 years. If fifteen years later another man died from bladder cancer at 60 years of age, he would have lost 22.1 years of life according to the 2005 male life table. As a result, his lifespan was shortened by 27%. The ALSS in the present study indicates an average expressed by percentage of lifespan shortened among all people who died from kidney or bladder cancer. Details of the ALSS measure and its utility have been described in our previous studies.^9^^,^^10^ All statistical analyses were performed using SAS 9.3 (SAS Institute Inc., Cary, NC, USA).

## RESULTS

Table 1 shows the number of deaths and ASRs due to kidney cancer in Japan from 1980 to 2010. Over a 30-year period, the actual number of deaths increased by about three-fold, from 866 deaths among men and 384 among women to 2,737 and 1,311 deaths among men and women, respectively. However, the ASRs among women were stable at around 0.6 per 100,000 women, and the rates slightly increased among men through the 1980s and 1990s, from 1.4 per 100,000 men to 1.9 per 100,000, and started slightly decreasing in the 2000s. We observed that over 20% of deaths occurred at 75 years or older in 1980 in both sexes, while the proportion in 2010 was 50.9% among men and 63.8% among women.

**Table 1.** Number of deaths, and age-standardized rate (ASR^a^) for deaths caused by kidney cancer in Japan, 1980–2010

Year	In men					In women				
	
	Distribution of deaths by age group, %			Total deaths	ASR	Distribution of deaths by age group, %			Total deaths	ASR
	
	<50	50–74	≥75			<50	50–74	≥75		
1980	12.2	65.7	22.1	866	1.4	12.8	65.9	21.4	384	0.5
1981	12.4	67.5	20.1	841	1.3	15.0	59.4	25.6	399	0.5
1982	11.9	69.1	19.0	975	1.5	17.3	58.0	24.8	440	0.5
1983	8.5	69.5	21.9	1,031	1.5	10.1	62.0	27.9	484	0.5
1984	8.7	69.2	22.1	1,137	1.6	11.2	54.4	34.4	491	0.5
1985	9.8	67.6	22.6	1,191	1.7	11.2	55.5	33.3	537	0.6
1986	9.1	66.6	24.3	1,241	1.7	8.9	54.1	37.0	597	0.6
1987	7.3	67.8	24.9	1,312	1.7	8.1	54.6	37.3	608	0.6
1988	7.1	66.3	26.6	1,366	1.7	8.0	51.3	40.7	634	0.6
1989	7.9	61.3	30.8	1,407	1.7	7.0	49.4	43.6	654	0.6
1990	7.6	62.6	29.7	1,547	1.8	7.2	49.9	43.0	726	0.6
1991	6.0	63.8	30.1	1,607	1.8	7.4	53.3	39.2	688	0.6
1992	8.5	62.0	29.6	1,678	1.8	6.6	49.3	44.1	758	0.6
1993	7.5	62.5	30.0	1,753	1.9	6.0	46.0	48.0	804	0.6
1994	6.8	62.5	30.6	1,855	1.9	6.7	45.7	47.6	845	0.6
1995	6.6	62.3	31.1	1,921	1.9	6.6	46.4	47.0	888	0.6
1996	6.4	62.2	31.5	2,006	1.9	8.2	42.6	49.2	951	0.7
1997	6.8	60.6	32.6	2,103	2.0	4.6	43.7	51.7	890	0.6
1998	6.1	59.8	34.0	2,127	1.9	6.2	46.2	47.6	1,000	0.7
1999	5.8	59.9	34.3	2,153	1.9	3.7	43.9	52.4	1,029	0.6
2000	4.8	62.1	33.1	2,134	1.8	5.5	39.8	54.7	1,028	0.6
2001	5.2	59.6	35.3	2,209	1.8	4.6	41.0	54.4	1,081	0.6
2002	5.0	57.3	37.7	2,409	1.9	3.3	40.8	56.0	1,138	0.6
2003	4.5	56.5	39.0	2,395	1.8	4.0	41.1	54.9	1,178	0.6
2004	4.7	57.6	37.7	2,491	1.9	3.6	41.2	55.2	1,244	0.7
2005	4.2	55.2	40.7	2,600	1.9	2.9	36.6	60.5	1,233	0.6
2006	3.9	55.7	40.4	2,498	1.8	4.3	36.6	59.1	1,269	0.6
2007	4.4	52.5	43.1	2,617	1.8	3.3	37.8	58.9	1,320	0.6
2008	4.0	50.9	45.1	2,649	1.8	3.5	32.3	64.2	1,274	0.6
2009	3.1	48.4	48.5	2,703	1.7	3.6	31.8	64.5	1,297	0.6
2010	3.2	45.9	50.9	2,737	1.7	3.4	32.7	63.8	1,311	0.6

^a^Age-standardized rate per 100,000 population according to the World Standard Population.

Table 2 provides information regarding bladder cancer deaths for the same period. We also observed about three-fold increases in the actual number of deaths over the study period. There were 1,606 deaths among men and 755 among women in 1980. The respective number of deaths increased to 4,719 among men and 2,085 among women in 2010. The ASRs for bladder cancer were somewhat stable among both sexes: around 2.4 or 2.5 per 100,000 men and around 0.6 or 0.7 per 100,000 women. We observed that higher proportions of bladder cancer deaths occurred at 75 years or older compared with those of kidney cancer. In 1980, 44.9% of deaths due to bladder cancer occurred in the oldest age group among men, and 52.3% of deaths due to bladder cancer among women, while these proportions increased to 71.3% and 81.9% among men and women, respectively.

**Table 2.** Number of deaths, and age-standardized rate (ASR^a^) for deaths caused by bladder cancer in Japan, 1980–2010

Year	In men					In women				
	
	Distribution of deaths by age group, %			Total deaths	ASR	Distribution of deaths by age group, %			Total deaths	ASR
	
	<50	50–74	≥75			<50	50–74	≥75		
1980	3.4	51.7	44.9	1,606	2.5	3.4	44.2	52.3	755	0.8
1981	3.5	50.7	45.9	1,615	2.5	1.9	44.1	54.0	732	0.8
1982	3.9	50.6	45.5	1,671	2.4	1.7	41.9	56.4	750	0.8
1983	4.0	48.0	48.0	1,670	2.4	2.7	39.0	58.4	826	0.8
1984	3.7	47.6	48.8	1,669	2.3	3.2	41.3	55.4	772	0.7
1985	2.9	47.6	49.5	1,705	2.3	2.4	40.3	57.3	872	0.8
1986	3.1	47.0	49.9	1,807	2.3	3.1	33.8	63.0	795	0.7
1987	2.6	44.8	52.5	1,815	2.2	1.7	32.6	65.6	806	0.6
1988	2.7	45.6	51.7	1,851	2.2	1.7	35.4	62.9	874	0.7
1989	1.6	45.1	53.3	1,999	2.3	1.9	30.6	67.5	939	0.7
1990	2.5	42.9	54.5	2,110	2.3	1.6	30.7	67.7	938	0.7
1991	3.0	41.9	55.2	2,165	2.3	2.6	29.1	68.4	1,008	0.7
1992	2.5	43.1	54.4	2,179	2.2	1.3	28.3	70.4	1,017	0.6
1993	2.3	42.2	55.5	2,217	2.2	2.0	28.5	69.5	1,062	0.6
1994	2.3	41.1	56.6	2,415	2.3	2.1	26.5	71.4	1,115	0.6
1995	2.5	39.2	58.3	2,700	2.5	1.3	25.7	73.0	1,231	0.7
1996	2.2	42.7	55.2	2,785	2.5	1.5	27.7	70.8	1,194	0.6
1997	1.8	40.9	57.3	2,856	2.4	1.7	24.8	73.5	1,277	0.6
1998	1.9	40.8	57.3	2,951	2.4	1.2	25.2	73.6	1,344	0.7
1999	1.6	40.2	58.2	3,296	2.6	1.2	23.2	75.6	1,485	0.7
2000	1.4	41.2	57.3	3,184	2.4	0.7	24.5	74.8	1,496	0.7
2001	1.6	38.4	59.9	3,458	2.5	1.1	23.1	75.8	1,587	0.7
2002	1.1	39.3	59.6	3,500	2.4	1.2	20.5	78.3	1,637	0.7
2003	1.4	36.6	62.0	3,719	2.5	1.2	22.3	76.5	1,693	0.7
2004	1.2	35.8	63.0	3,817	2.4	0.7	21.7	77.6	1,738	0.7
2005	1.3	34.2	64.5	4,141	2.5	0.6	20.3	79.0	1,888	0.7
2006	0.9	32.4	66.7	4,216	2.5	0.8	20.3	78.8	1,909	0.7
2007	1.1	31.7	67.2	4,271	2.4	0.8	20.2	78.9	1,903	0.6
2008	1.0	30.6	68.5	4,438	2.4	0.7	18.9	80.4	2,029	0.6
2009	1.0	29.4	69.7	4,478	2.3	0.7	15.9	83.4	2,147	0.6
2010	0.7	28.0	71.3	4,719	2.4	0.8	17.4	81.9	2,085	0.6

^a^Age-standardized rate per 100,000 population according to the World Standard Population.

Table 3 shows information on the total YLL, adjusted YLL rates per 1,000 individuals, AYLL per decedent, and ALSS among decedents for kidney cancer. The total YLL increased according to increases in number of deaths over time, and the adjusted YLL rates slightly increased over the study period for both sexes. We observed that AYLL decreased from 16.9 to 13.3 years of life among men and from 20.6 to 14.5 years among women in this period. We also observed a favorable trend of ALSS, indicating that men died 21.0% earlier than the expected lifespan in 1980 and 15.3% in 2010. Among women, the lifespan was on average shortened by 24.7% in 1980 and 15.8% in 2010.

**Table 3.** Total years of life lost (YLL), adjusted YLL rate^a^, average years of life lost (AYLL), and average lifespan shortened (ALSS) due to kidney cancer in Japan, 1980–2010

Year	In men							In women						
	
	Distribution of YLL by age group, %			Total YLL, years	Adjusted rate of YLL per 1,000 men	AYLL per decedent, years	ALSS among decedents, %	Distribution of YLL by age group, %			Total YLL, years	Adjusted rate of YLL per 1,000 women	AYLL per decedent, years	ALSS among decedents, %
	
	<50	50–74	≥75					<50	50–74	≥75				
1980	28.4	63.3	8.2	14,637	0.24	16.9	21.0	32.8	59.3	7.8	7,926	0.12	20.6	24.7
1981	28.1	64.3	7.7	14,043	0.23	16.7	20.8	36.9	53.5	9.6	8,070	0.12	20.2	24.2
1982	26.8	66.1	7.0	16,458	0.26	16.9	21.0	40.3	51.1	8.6	9,149	0.14	20.8	24.8
1983	19.5	71.7	8.8	16,176	0.24	15.7	19.5	25.5	63.5	11.0	8,655	0.11	17.9	21.2
1984	21.4	69.8	8.8	18,024	0.28	15.9	19.6	29.6	56.5	13.8	8,809	0.12	17.9	21.3
1985	22.6	68.5	8.9	20,100	0.30	16.9	20.7	29.1	57.3	13.6	10,234	0.13	19.1	22.3
1986	21.5	68.8	9.6	20,339	0.29	16.4	20.1	22.7	61.1	16.2	10,387	0.12	17.4	20.3
1987	18.0	71.8	10.2	21,158	0.29	16.1	19.7	22.6	61.1	16.2	10,643	0.13	17.5	20.4
1988	17.9	71.1	11.0	21,115	0.28	15.5	18.8	21.8	59.8	18.3	10,572	0.12	16.7	19.4
1989	19.0	68.4	12.7	21,557	0.27	15.3	18.6	19.9	60.4	19.7	10,503	0.12	16.1	18.6
1990	17.8	69.6	12.6	24,648	0.30	15.9	19.2	20.7	59.5	19.8	12,554	0.14	17.3	19.8
1991	15.2	72.0	12.8	25,220	0.31	15.7	18.9	20.1	62.2	17.7	11,956	0.13	17.4	20.0
1992	20.2	67.5	12.3	26,447	0.32	15.8	19.0	19.1	59.9	20.9	12,660	0.14	16.7	19.1
1993	17.7	69.8	12.5	26,900	0.31	15.3	18.5	18.1	58.9	23.0	12,632	0.13	15.7	17.9
1994	16.2	71.0	12.8	28,069	0.31	15.1	18.2	18.2	59.6	22.2	13,178	0.13	15.6	17.8
1995	15.9	70.8	13.4	28,925	0.31	15.1	18.0	17.6	59.5	22.9	14,520	0.13	16.4	18.5
1996	15.5	71.2	13.3	30,180	0.32	15.0	18.0	22.8	53.0	24.1	15,705	0.15	16.5	18.6
1997	16.3	69.7	14.0	31,467	0.33	15.0	17.9	14.0	60.3	25.7	13,695	0.13	15.4	17.3
1998	15.7	69.3	15.1	31,036	0.32	14.6	17.4	17.2	60.3	22.5	16,383	0.15	16.4	18.4
1999	14.3	70.4	15.3	30,957	0.31	14.4	17.1	10.9	62.5	26.6	15,345	0.12	14.9	16.7
2000	11.4	72.9	15.7	32,468	0.31	15.2	17.9	17.0	54.1	28.9	16,619	0.15	16.2	17.9
2001	13.1	70.5	16.5	34,013	0.32	15.4	18.1	13.7	57.6	28.7	16,975	0.14	15.7	17.3
2002	12.6	69.5	17.8	35,900	0.33	14.9	17.5	9.7	59.5	30.8	17,401	0.13	15.3	16.9
2003	11.6	68.9	19.5	34,932	0.31	14.6	17.1	12.9	58.0	29.1	18,442	0.15	15.7	17.3
2004	11.6	70.2	18.2	37,413	0.33	15.0	17.6	11.2	59.7	29.1	19,651	0.16	15.8	17.4
2005	11.3	68.9	19.8	38,643	0.34	14.9	17.3	9.5	56.1	34.4	18,525	0.13	15.0	16.4
2006	10.3	69.5	20.2	37,293	0.33	14.9	17.4	13.4	54.5	32.2	20,084	0.16	15.8	17.3
2007	11.5	67.1	21.5	37,932	0.32	14.5	16.8	10.7	57.4	31.9	20,379	0.15	15.4	16.9
2008	11.2	65.8	23.0	37,669	0.32	14.2	16.5	11.5	51.3	37.2	18,330	0.13	14.4	15.7
2009	8.5	65.5	26.0	36,853	0.29	13.6	15.8	13.2	49.2	37.6	19,058	0.15	14.7	16.0
2010	9.6	63.2	27.3	36,294	0.28	13.3	15.3	11.7	52.2	36.1	19,038	0.14	14.5	15.8

^a^Adjusted YLL rate per 1,000 population according to the World Standard Population.

Table 4 shows relevant information on respective measures of premature mortality for bladder cancer. We observed increases in the total YLL in both sexes, as well as slight increases of the adjusted YLL rates over the study period among men. We observed that AYLL decreased by about 2 years of life, from 11.2 to 9.6 years among men and from 12.3 to 10.4 years among women at the end of this study period. Results of the ALSS measure show that patients lost a smaller proportion of their life at the end of the period, decreasing from 13.5% of their life in 1980 to 10.8% in 2010 among men and from 14.2% of the expected life to 11.1% among women.

**Table 4.** Total years of life lost (YLL), adjusted YLL rate^a^, average years of life lost (AYLL), and average lifespan shortened (ALSS) due to bladder cancer in Japan, 1980–2010

Year	In men							In women						
	
	Distribution of YLL by age group, %			Total YLL, years	Adjusted rate of YLL per 1,000 men	AYLL per decedent, years	ALSS among decedents, %	Distribution of YLL by age group, %			Total YLL, years	Adjusted rate of YLL per 1,000 women	AYLL per decedent, years	ALSS among decedents, %
	
	<50	50–74	≥75					<50	50–74	≥75				
1980	9.7	66.6	23.6	18,053	0.28	11.2	13.5	10.4	61.1	28.5	9,261	0.11	12.3	14.2
1981	10.4	65.3	24.3	17,857	0.27	11.1	13.3	6.9	62.8	30.3	8,683	0.10	11.9	13.7
1982	12.2	64.5	23.3	18,958	0.28	11.3	13.6	6.3	60.8	32.9	8,665	0.10	11.6	13.4
1983	12.0	62.9	25.2	18,398	0.26	11.0	13.2	9.2	58.0	32.8	9,350	0.10	11.3	13.0
1984	11.2	63.4	25.4	18,179	0.25	10.9	13.0	11.0	58.2	30.7	9,075	0.10	11.8	13.6
1985	8.7	64.6	26.7	19,221	0.26	11.3	13.4	7.7	58.9	33.5	10,682	0.11	12.2	14.0
1986	9.8	63.9	26.3	20,244	0.27	11.2	13.2	10.9	51.2	37.8	9,416	0.09	11.8	13.5
1987	8.5	63.4	28.1	20,069	0.25	11.1	13.1	7.3	53.3	39.5	8,944	0.09	11.1	12.6
1988	8.6	64.1	27.3	20,201	0.25	10.9	12.9	6.1	56.2	37.6	9,807	0.09	11.2	12.7
1989	5.2	65.6	29.1	20,976	0.25	10.5	12.3	7.5	50.8	41.7	9,913	0.09	10.6	11.9
1990	8.2	62.1	29.7	23,256	0.27	11.0	12.9	5.9	50.5	43.5	10,550	0.09	11.2	12.6
1991	9.6	60.1	30.3	23,820	0.27	11.0	12.8	9.2	48.6	42.3	11,572	0.10	11.5	12.8
1992	8.0	62.4	29.7	23,941	0.26	11.0	12.8	5.1	49.3	45.6	10,998	0.08	10.8	12.1
1993	7.1	62.4	30.5	23,891	0.25	10.8	12.6	7.5	47.8	44.8	11,524	0.09	10.9	12.1
1994	7.5	61.2	31.4	25,709	0.26	10.6	12.4	7.8	46.7	45.5	12,134	0.09	10.9	12.1
1995	8.0	59.3	32.8	28,441	0.28	10.5	12.2	4.9	45.8	49.2	13,433	0.09	10.9	12.0
1996	7.0	62.1	30.9	29,866	0.28	10.7	12.4	5.6	49.3	45.1	13,396	0.09	11.2	12.4
1997	6.0	61.6	32.3	29,334	0.27	10.3	11.8	6.5	45.2	48.3	13,695	0.09	10.7	11.8
1998	6.3	61.7	32.0	30,508	0.27	10.3	11.9	4.8	46.2	49.0	14,551	0.09	10.8	11.9
1999	5.4	61.7	32.8	33,974	0.29	10.3	11.9	4.9	43.8	51.3	15,271	0.09	10.3	11.3
2000	4.6	61.8	33.6	35,403	0.29	11.1	12.7	2.8	45.9	51.3	17,203	0.10	11.5	12.5
2001	5.5	58.8	35.8	37,708	0.31	10.9	12.4	4.3	43.5	52.2	17,600	0.10	11.1	12.0
2002	3.8	60.2	36.1	37,865	0.29	10.8	12.3	4.9	39.8	55.3	17,881	0.10	10.9	11.8
2003	4.6	57.0	38.4	40,206	0.31	10.8	12.3	5.2	42.0	52.9	18,840	0.11	11.1	12.0
2004	4.3	56.2	39.5	40,293	0.30	10.6	12.0	2.7	42.7	54.6	18,678	0.10	10.7	11.6
2005	4.4	54.9	40.7	44,636	0.32	10.8	12.2	2.5	40.5	57.0	20,457	0.10	10.8	11.6
2006	3.0	54.0	43.0	43,957	0.30	10.4	11.8	3.4	39.8	56.7	20,799	0.10	10.9	11.7
2007	4.0	52.4	43.6	44,386	0.30	10.4	11.7	3.8	39.9	56.4	20,996	0.11	11.0	11.9
2008	3.6	51.8	44.7	45,607	0.30	10.3	11.6	3.1	37.9	59.0	21,601	0.10	10.6	11.4
2009	3.7	50.7	45.6	44,707	0.28	10.0	11.2	2.9	33.5	63.6	21,677	0.09	10.1	10.8
2010	2.6	50.0	47.4	45,479	0.28	9.6	10.8	3.3	36.4	60.3	21,636	0.10	10.4	11.1

^a^Adjusted YLL rate per 1,000 population according to the World Standard Population.

## DISCUSSION

To the best of our knowledge, the present study is the first comprehensive analysis of long-term trends in premature mortality due to kidney and bladder cancer among the entire Japanese population. The results showed that, on average, the lifespan of patients was prolonged by about 6 percentage points among men and 9 percentage points among women for kidney cancer and by about 3 percentage points among both sexes for bladder cancer. In other words, patients with kidney and bladder cancers died at older ages at the end of study period.

In Japan, malignancies of the urinary tract are less frequently diagnosed compared with other leading cancer sites, like stomach, lung, breast (among women), colorectal, and liver.^2^ In the present analysis, we included deaths from the two most predominant subtypes of urinary tract cancers. Our results show that the number of deaths and ASRs were consistently higher for bladder than kidney cancers. Over the study period, the number of deaths increased by over three-fold for kidney and bladder cancers. However, the ASRs did not increase as much; rather, they were almost stable over the study period.

Given that incidence and mortality rates do not fully reflect the burden of loss due to cancer deaths, two common “life lost” measures, YLL and AYLL, have been used for depicting premature mortality. The former indicates a total burden as a whole among the entire population and is proportional to the number of deaths, while the latter indicates the average of the burden per decedent. The increase in terms of the total YLL is in accordance with the increased number of deaths over time that has been a consequence of population growth and aging of the entire Japanese population. Indeed, the Japanese population grew from 57 to 61 million men and from 59 to 64 million women over the study period. However, the AYLL measure shows that, on average, people with kidney and bladder cancer in Japan lost smaller proportions of their lives at the end of the study period. Unlike favorable trends of the AYLL, the adjusted YLL rates over time among the entire population slightly increased for kidney cancers and were stable for bladder cancers.

Having already discussed,^9^^,^^10^^,^^13^ it is worth mentioning that there are two major algorithms in YLL calculation: the threshold and the life table method. According to the threshold method, only deaths that occurred before an arbitrary limit were considered premature. Indeed, our Table 2 shows that 45% and 71% of bladder cancer deaths occurred among men aged at 75 years or older in 1980 and 2010, respectively. In the other words, there were a substantial number of deaths disregarded in YLL calculation if a cut-off at 75 years was applied. In contrast, the YLL calculation in our study applied the life table method, which was based on a comparison of age at death and respective life expectancy according to the national life tables. The life table method considers all deaths at any age in the calculation. In the present study, favorable trends of premature mortality were supported by the fact the deaths occurred gradually at older ages over the study period (see Table 1 and Table 2). We observed that proportions of those who died at less than 50 years of age decreased, while those who died at 75 years or older increased at the end of the study. Given the differences in YLL calculation, caution should be exercised when attempting to compare results from different studies.

We have for the first time introduced the conceptualization of a novel ALSS measure for premature mortality in our previous studies.^9^^,^^10^ This measure is expressed as a percentage of their life shortened in relation to their expected lifespan if patients would not die early due to a given chronic condition. The expected lifespan is calculated according to respective national sex-specific life tables. We have demonstrated that the utility of the measure is more intuitive, especially for clinicians and health policy makers, without the need to further take into account changes in the national life table structure when interpreting the findings.^9^^,^^10^ In the present study, although both AYLL and ALSS measures show favorable trends, interpretation of the AYLL results often requires a consideration of change of the life tables over time, whereas that of the ALSS results does not. For instance, the AYLL measure in Table 3 shows that, on average, men died of kidney cancer at 16.9 years earlier than expectation in 1980, while they lost 13.3 years of life at the end of the study. However, the absolute improvement would be about 9 years of life since life expectancy at birth actually increased by 6 years (from 73 to 79 years) over the study period. Indeed, the changes in life expectancy over time also affected ALSS calculation. The two hypothetical examples that we provided in the method section implied that the estimate of ALSS would result in a longer proportion of lifespan lost when life expectancy is longer. In contrast, the favorable trends of ALSS for kidney or bladder cancer indicated that the lifespan of patients was prolonged faster than the general population experienced over the study period. In this study, the ALSS measure indicates that men with kidney cancer, for instance, lost an average of 21% of their lifespan at the beginning of the study period, and they lost an average of 15% of their lifespan at the end of the study. Hence, their lifespans were prolonged by about 6 percentage points over a 30-year period. In addition, the measure indicates that patients with bladder cancer lost, on average, a smaller proportion of life compared with kidney patients. Indeed, men with bladder cancer lost only about 11% of their lifespan in 2010, compared with kidney patients whose ALSS was 15% for the same year. Hence, the development of this new ALSS measure contributes to the monitoring of premature mortality over time and allows comparison between chronic health conditions.

Renal ultrasonography is a useful tool to detect early stage kidney cancer.^14^ Mihara et al^15^ reported that abdominal ultrasonographic screening of 219,640 people had been performed in Japan and 723 cases of malignant tumors were detected, of which kidney cancer accounted for 26.6% of the cases. In all cases of kidney cancer, no symptoms were noticed and no abnormalities were found in the blood chemistry tests or urinalysis. With respect to pathological stage, 89% of the tumors were ≤pT2. Although radical nephrectomy has traditionally been performed in patients with localized kidney cancer, partial nephrectomy represents the current standard of care for T1 kidney cancers that are 4 cm or less in diameter in the past decade.^16^ The standard treatment for advanced kidney cancers has changed dramatically in the past decade, from cytokine therapy to molecular-targeted therapy. Shinohara et al^17^ reported that the median overall survival was 27.2 months in Japanese patients with metastatic kidney cancer in the era of molecular-targeted therapy. Before introduction of molecular-targeted therapy, the median overall survival time for metastatic renal cell carcinoma was 21.4 months, as reported in a study conducted among 1,463 patients during 1988 to 2002.^18^ Therefore, the newly approved molecular-targeted therapy might favorably contribute to future trends of premature mortality for kidney cancer. In contrast, over the past 2 decades, patients with metastatic bladder cancer had only limited therapeutic options following cisplatin-based chemotherapy (methotrexate, vinblastine, doxorubicin, and cisplatin or gemcitabine plus cisplatin). Cisplatin-based chemotherapy conferred a median overall survival of 12 to 15 months.^19^ In addition, while bladder tumors superficial to the muscularis propria are effectively treated with transurethral resection and intravesical therapy, there is a significant rate of recurrence and poor prognosis after radical cystectomy for muscle-invasive bladder cancer.^20^ Lastly, it is possible that more kidney and bladder cancer cases, diagnosed recently among elderly individuals due to rapid aging of the entire Japanese population, might also support the favorable trends of premature mortality observed in this study. Nevertheless, the aforementioned changes in diagnosis and treatment practice over the last decades may have contributed to improvement of premature mortality for both kidney and bladder cancer in Japan.

The strength of this study was the introduction of a new measure, ALSS, in addition to the existing measures of premature mortality. Moreover, the new measure is more comparable over time, allowing us to show comprehensive long-term trends of premature mortality over a 30-year period. An additional strength of the current analysis was the robust and complete nature of the vital statistics data because death notification is required in Japan. A limitation of the study warrants mentioning: the nature of the study design did not allow us to directly investigate the causal relationship between the improvement in premature mortality and contributing factors like diagnosis, treatments, and/or follow-up. Rather, this study merely provided a descriptive analysis, so any causal inference of the findings should be interpreted with caution.

In summary, results from this study show that patients with malignancies of the urinary system have had their lives prolonged over a 30-year period in Japan. The favorable trends in premature mortality may be a proxy indicator of how effectively the health care system has functioned with respect to diagnosis and treatment of kidney and bladder cancer in Japan over the study period.

## References

[1] Parkin DM, Bray F, Ferlay J, Pisani P Global cancer statistics, 2002. CA Cancer J Clin. 2005;55:74–108. 10.3322/canjclin.55.2.74 15761078

[2] Marugame T, Kamo K, Katanoda K, Ajiki W, Sobue T Cancer incidence and incidence rates in Japan in 2000: Estimates based on data from 11 population-based cancer registries. Jpn J Clin Oncol. 2006;36:668–675. 10.1093/jjco/hyl084 16926226

[3] Arcà M, di Orio F, Forastiere F, Tasco C, Perucci CA Years of potential life lost (YPLL) before age 65 in Italy. Am J Public Health. 1988;78:1202–1205. 10.2105/AJPH.78.9.1202 3407820 PMC1349394

[4] Murray CJ, Lopez AD. *The Global Burden of Disease: a comprehensive assessment of mortality and disability from diseases, injuries, and risk factors in 1990 and projected to 2020*. Boston: Harvard University Press, World Health Organization, Harvard School of Public Health, World Bank; 1996.

[5] Murray CJ, Lopez AD. *The Global Health Statistics: a compendium of incidence, prevalence and mortality estimates for over 200 conditions*. Boston: Harvard University Press, World Health Organization, Harvard School of Public Health, World Bank; 1996.

[6] Murray CJ, Lopez AD Alternative projections of mortality and disability by cause 1990–2020: Global Burden of Disease Study. Lancet. 1997;349:1498–1504. 10.1016/S0140-6736(96)07492-2 9167458

[7] Global Burden of Disease Study 2013 Collaborators Global, regional, and national incidence, prevalence, and years lived with disability for 301 acute and chronic diseases and injuries in 188 countries, 1990–2013: a systematic analysis for the Global Burden of Disease Study 2013. Lancet. 2015;386:743–800. 10.1016/S0140-6736(15)60692-4 26063472 PMC4561509

[8] Murray CJ, Lopez AD Mortality by cause for eight regions of the world: Global Burden of Disease Study. Lancet. 1997;349:1269–1276. 10.1016/S0140-6736(96)07493-4 9142060

[9] Pham TM, Sikdar KC, Kaposhi B, Lupichuk S, Yang H, Shack L Premature mortality due to breast cancer among Canadian women: an analysis of a 30-year period from 1980 through 2010. Eur J Public Health. 2018;28:348–352. 10.1093/eurpub/ckx195 29112730

[10] Pham TM, Sikdar KC, Cheung WY, Premature mortality due to malignancies of the central nervous system in Canada, 1980–2010. Neuroepidemiology. 2018;50:195–200. 10.1159/000488145 29694962

[11] World Health Organization. WHO statistical information system. WHO mortality database. Available from: http://www.who.int/healthinfo/mortality_data/en/. Accessed: September 18, 2017.

[12] Statistics and Information Department, Minister’s Secretariat, Ministry of Health and Welfare, Japanese Government. Available from: http://www.mhlw.go.jp/english/database/db-hw/vs02.html. Accessed: November 10, 2017.

[13] Pham TM, Fujino Y, Matsuda S, Yoshimura T Premature mortality due to cancer in Japan, 1995 and 2005. Int J Cancer. 2010;127:190–194. 10.1002/ijc.25021 19882711

[14] Tosaka A, Ohya K, Yamada K, Incidence and properties of renal masses and asymptomatic renal cell carcinoma detected by abdominal ultrasonography. J Urol. 1990;144:1097–1099. 10.1016/S0022-5347(17)39667-2 2231878

[15] Mihara S, Kuroda K, Yoshioka R, Koyama W Early detection of renal cell carcinoma by ultrasonographic screening—based on the results of 13 years screening in Japan. Ultrasound Med Biol. 1999;25:1033–1039. 10.1016/S0301-5629(99)00070-8 10574334

[16] Ljungberg B, Bensalah K, Canfield S, EAU guidelines on renal cell carcinoma: 2014 update. Eur Urol. 2015;67:913–924. 10.1016/j.eururo.2015.01.005 25616710

[17] Shinohara N, Obara W, Tatsugami K, Prognosis of Japanese patients with previously untreated metastatic renal cell carcinoma in the era of molecular-targeted therapy. Cancer Sci. 2015;106:618–626. 10.1111/cas.12646 25711777 PMC4452164

[18] Naito S, Yamamoto N, Takayama T, Prognosis of Japanese metastatic renal cell carcinoma patients in the cytokine era: a cooperative group report of 1,463 patients. Eur Urol. 2010;57:317–325. 10.1016/j.eururo.2008.12.026 19136199

[19] Seront E, Machiels JP Molecular biology and targeted therapies for urothelial carcinoma. Cancer Treat Rev. 2015;41:341–353. 10.1016/j.ctrv.2015.03.004 25828962

[20] Grossman HB, Natale RB, Tangen CM, Neoadjuvant chemotherapy plus cystectomy compared with cystectomy alone for locally advanced bladder cancer. N Engl J Med. 2003;349:859–866. 10.1056/NEJMoa022148 12944571

